# 3-Allyl-6-bromo-1*H*-imidazo[4,5-*b*]pyridin-2(3*H*)-one

**DOI:** 10.1107/S1600536811025037

**Published:** 2011-07-09

**Authors:** Siham Dahmani, Youssef Kandri Rodi, Santiago V. Luis, Michael Bolte, Lahcen El Ammari

**Affiliations:** a Laboratoire de Chimie Organique Appliquée, Université Sidi Mohamed Ben Abdallah, Faculté des Sciences et Techniques, Route d’Immouzzer, BP 2202 Fès, Morocco; b Departamento de Quimica Inorganica & Organica, ESTCE, Universitat Jaume I, E-12080 Castellon, Spain; c Institut für Anorganische Chemie, J. W. Goethe-Universität Frankfurt, Max-von-Laue-Strasse 7, 60438 Frankfurt/Main, Germany; d Laboratoire de Chimie du Solide Appliquée, Faculté des Sciences, Université Mohammed V-Agdal, Avenue Ibn Battouta, BP 1014, Rabat, Morocco

## Abstract

In the mol­ecule of the title compound, C_9_H_8_BrN_3_O, the fused-ring system is almost planar, the largest deviation from the mean plane being 0.008 (3) Å. The plane through the atoms forming the allyl group is roughly perpendicular to the imidazo[4,5-*b*]pyridin-2-one system, as indicated by the dihedral angle between them of 70.28 (11)°. In the crystal, each mol­ecule is linked to its symmetry equivalent about the center of inversion by a pair of strong N—H⋯O hydrogen bond, forming inversion dimers.

## Related literature

For background to the biological activity of imidazopyridines, see: Chen & Dost (1992 ▶); Cappelli *et al.* (2006 ▶); Weier *et al.* (1993 ▶, 1994 ▶); Kulkarni & Newman (2007 ▶). For background to their pharmacological activity, see: Bavetsias *et al.* (2007 ▶, 2010 ▶). 
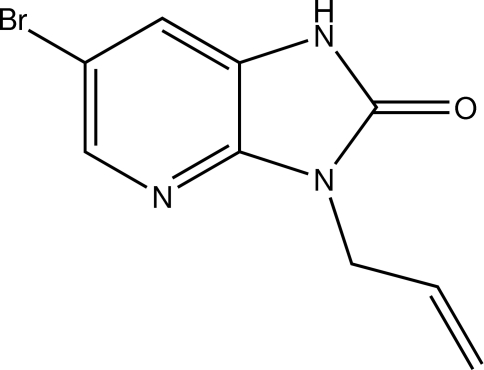


## Experimental

### 

#### Crystal data

C_9_H_8_BrN_3_O
*M* *_r_* = 254.09
Triclinic, 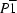

*a* = 4.5138 (5) Å
*b* = 9.7750 (9) Å
*c* = 11.5717 (11) Å
α = 78.748 (2)°
β = 82.526 (3)°
γ = 86.038 (2)°
*V* = 496.00 (9) Å^3^
*Z* = 2
Mo *K*α radiation
μ = 4.11 mm^−1^
*T* = 571 K
0.60 × 0.19 × 0.04 mm

#### Data collection

Bruker CCD three-circle diffractometer
Absorption correction: multi-scan (*SADABS*; Sheldrick, 1996 ▶) *T*_min_ = 0.192, *T*_max_ = 0.850
3086 measured reflections
2019 independent reflections
1683 reflections with *I* > 2σ(*I*)
*R*_int_ = 0.026

#### Refinement

*R*[*F* ^2^ > 2σ(*F* ^2^)] = 0.046
*wR*(*F* ^2^) = 0.127
*S* = 1.09
2019 reflections
127 parameters
H-atom parameters constrained
Δρ_max_ = 0.89 e Å^−3^
Δρ_min_ = −0.82 e Å^−3^

### 

Data collection: *SMART* (Bruker, 1997 ▶); cell refinement: *SAINT* (Bruker, 1997 ▶); data reduction: *SAINT*; program(s) used to solve structure: *SHELXS97* (Sheldrick, 2008 ▶); program(s) used to refine structure: *SHELXL97* (Sheldrick, 2008 ▶); molecular graphics: *ORTEP-3 for Windows* (Farrugia, 1997 ▶); software used to prepare material for publication: *WinGX* (Farrugia, 1999 ▶).

## Supplementary Material

Crystal structure: contains datablock(s) I, global. DOI: 10.1107/S1600536811025037/sj5172sup1.cif

Structure factors: contains datablock(s) I. DOI: 10.1107/S1600536811025037/sj5172Isup2.hkl

Supplementary material file. DOI: 10.1107/S1600536811025037/sj5172Isup3.cml

Additional supplementary materials:  crystallographic information; 3D view; checkCIF report

## Figures and Tables

**Table 1:** Hydrogen-bond geometry (Å, °)

*D*—H⋯*A*	*D*—H	H⋯*A*	*D*⋯*A*	*D*—H⋯*A*
N1—H1⋯O1^i^	0.86	1.95	2.798 (4)	168

Symmetry code: (i) 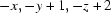.

**Fig. 1.**
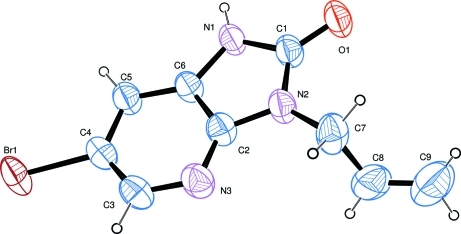
: Plot of the molecules of the title compound with the atom-labelling scheme. Displacement ellipsoids are drawn at the 50% probability level. H atoms are represented as small circles.

**Fig. 2.**
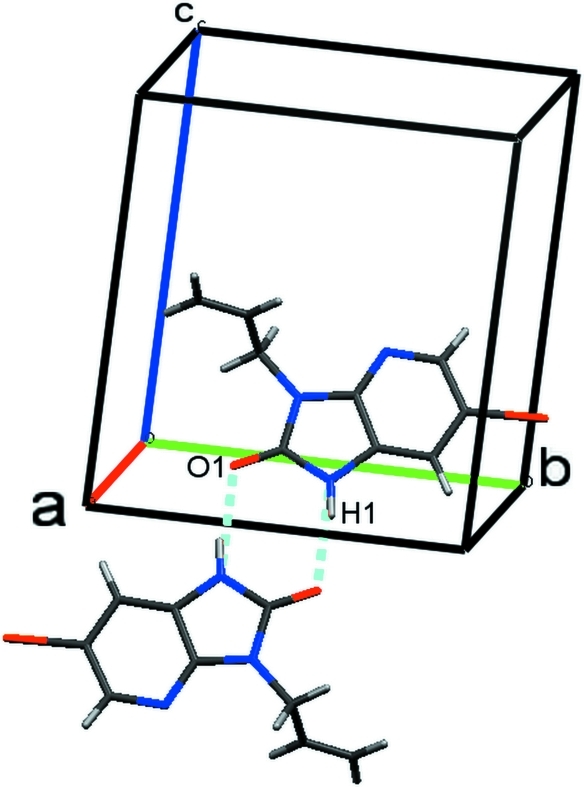
Partial plot showing N–H···O hydrogen bonds (dashed lines) between two symmetrical molecules (pseudo-dimer).

## supplementary crystallographic information

### Comment

Imidazopyridine molecules are important pharmacophores, which have proven to be useful for a number of biologically relevant targets. The compounds derived from the imidazopyridine system have recently been evaluated as antagonists of various biological receptors, including angiotensin-II (Chen & Dost 1992; Cappelli *et al.*, 2006), platelet activating factor (Weier *et al.*, (1993, 1994), and metabotropic glutamate subtype V (Kulkarni & Newman, 2007). Recently, a series of imidazo[4,5-*b*] pyridine derivatives as orally bioavailable Aurora A inhibitors with excellent potencies were reported (Bavetsias *et al.*, 2007, 2010). Hence, the synthesis of imidazo [4,5-*b*]pyridine derivatives is currently of great interest. Despite the importance of these intermediates, the methodology available for the synthesis was generally target-specific and restrictive in its scope.

Here, we wish to report a novel route leading to 3-allyl-6-bromo-1,3-dihydro- imidazo[4,5-*b*]pyridin-2-one. We have checked the action of allyllbromide towards 6-bromo-1,3-dihydro-imidazo[4,5 - b-]pyridin-2- one using K_2_CO_3_ as a base (scheme 1).

The two fused five and six-membered rings building the molecule are nearly planar with the maximum deviation of 0.008 (3)Å from C1 (Fig. 1). The dihedral angle between the imidazo[4,5-*b*]pyridin-2-one system and the plane through the atoms forming the allyl group is about 70.28 (11)°. The allyl group is nearly perpendicular to the imidazo[4,5-*b*]pyridin-2-one system and the torsion angle N2–C7–C8–C9 is in the range of -131.6 (5)°. In the crystal, each molecule is linked to its symmetry equivalent about an inversion center by a strong N—H···O hydrogen bond to form a pseudo dimers as shown in Fig.2 and Table 1.

### Experimental

To a stirred solution of 6-bromo-1,3-dihydro-imidazo[4,5 - b-]pyridin-2-one (0.2 g; 93.4 mmol), K_2_CO_3_ (0.38 g; 2.8 mmol), and tetra *n*-butyl ammonium bromide (0.03 g; 9.34 10–5 mol)in DMF, allyllbromide (0.097 ml; 1.12 mmol) was added dropwise. Later the mixture was heated under reflux for 24 h. After completion of reaction (monitored by TLC), the salt was filtered and the solvent was removed under reduced pressure. The resulting residue was purified by column chromatography on silica gel using (ethylacetate/hexane) (1/1) as eluent.  The crystals were obtained by dissolving 80 mg of product in 4 mL of methanol at  about 353 K, followed by a slow evaporation of the solvent.

### Refinement

H atoms were located in a difference map and treated as riding with C—H = 0.93 Å, 0.97, Å, and 0.86 Å for aromatic, methylene and –NH * respectively. All H atoms had *U*_iso_(H) = 1.2 *U*_eq_ (aromatic, methylene, –NH).

### Crystal data

**Table:**

C_9_H_8_BrN_3_O	*Z* = 2
*M**_r_* = 254.09	*F*(000) = 252
Triclinic, *P*1	*D*_x_ = 1.701 Mg m^−^^3^
Hall symbol: -P 1	Mo *K*α radiation, λ = 0.71073 Å
*a* = 4.5138 (5) Å	Cell parameters from 2019 reflections
*b* = 9.7750 (9) Å	θ = 1.8–26.4°
*c* = 11.5717 (11) Å	µ = 4.11 mm^−^^1^
α = 78.748 (2)°	*T* = 571 K
β = 82.526 (3)°	Fiber, colourless
γ = 86.038 (2)°	0.60 × 0.19 × 0.04 mm
*V* = 496.00 (9) Å^3^	

### Data collection

**Table:**

Bruker CCD three-circle diffractometer	2019 independent reflections
Radiation source: fine-focus sealed tube	1683 reflections with *I* > 2σ(*I*)
graphite	*R*_int_ = 0.026
ω scans	θ_max_ = 26.4°, θ_min_ = 1.8°
Absorption correction: multi-scan (*SADABS*; Sheldrick, 1996)	*h* = −5→5
*T*_min_ = 0.192, *T*_max_ = 0.850	*k* = −12→11
3086 measured reflections	*l* = −12→14

### Refinement

**Table:**

Refinement on *F*^2^	Primary atom site location: structure-invariant direct methods
Least-squares matrix: full	Secondary atom site location: difference Fourier map
*R*[*F*^2^ > 2σ(*F*^2^)] = 0.046	Hydrogen site location: inferred from neighbouring sites
*wR*(*F*^2^) = 0.127	H-atom parameters constrained
*S* = 1.09	*w* = 1/[σ^2^(*F*_o_^2^) + (0.0649*P*)^2^ + 0.1269*P*] where *P* = (*F*_o_^2^ + 2*F*_c_^2^)/3
2019 reflections	(Δ/σ)_max_ < 0.001
127 parameters	Δρ_max_ = 0.89 e Å^−^^3^
0 restraints	Δρ_min_ = −0.82 e Å^−^^3^

### Special details

**Table:**

Geometry. All e.s.d.'s (except the e.s.d. in the dihedral angle between two l.s. planes) are estimated using the full covariance matrix. The cell e.s.d.'s are taken into account individually in the estimation of e.s.d.'s in distances, angles and torsion angles; correlations between e.s.d.'s in cell parameters are only used when they are defined by crystal symmetry. An approximate (isotropic) treatment of cell e.s.d.'s is used for estimating e.s.d.'s involving l.s. planes.
Refinement. Refinement of *F*^2^ against all reflections. The weighted *R*-factor *wR* and goodness of fit *S* are based on *F*^2^, conventional *R*-factors *R* are based on *F*, with *F* set to zero for negative *F*^2^. The threshold expression of *F*^2^ > σ(*F*^2^) is used only for calculating *R*-factors(gt) *etc*. and is not relevant to the choice of reflections for refinement. *R*-factors based on *F*^2^ are statistically about twice as large as those based on *F*, and *R*- factors based on all data will be even larger.

### Fractional atomic coordinates and isotropic or equivalent isotropic displacement parameters (Å^2^)

**Table:**

	*x*	*y*	*z*	*U*_iso_*/*U*_eq_	
Br1	0.99854 (9)	−0.03225 (4)	0.83292 (4)	0.0624 (2)	
N1	0.3023 (6)	0.4132 (3)	0.9218 (3)	0.0429 (6)	
H1	0.1781	0.3823	0.9827	0.052*	
C1	0.3081 (8)	0.5487 (4)	0.8627 (3)	0.0438 (7)	
O1	0.1416 (6)	0.6472 (3)	0.8854 (2)	0.0545 (6)	
N2	0.5408 (7)	0.5549 (3)	0.7722 (3)	0.0463 (7)	
C2	0.6765 (8)	0.4238 (4)	0.7739 (3)	0.0448 (8)	
N3	0.9047 (7)	0.3902 (3)	0.7003 (3)	0.0536 (7)	
C3	0.9924 (8)	0.2536 (4)	0.7221 (4)	0.0537 (9)	
H3	1.1527	0.2227	0.6733	0.064*	
C4	0.8551 (8)	0.1578 (4)	0.8137 (3)	0.0474 (8)	
C5	0.6169 (8)	0.1954 (3)	0.8914 (3)	0.0446 (7)	
H5	0.5260	0.1316	0.9541	0.054*	
C6	0.5261 (7)	0.3327 (3)	0.8692 (3)	0.0400 (7)	
C7	0.6297 (9)	0.6836 (4)	0.6904 (4)	0.0577 (10)	
H7A	0.8450	0.6793	0.6698	0.069*	
H7B	0.5786	0.7622	0.7302	0.069*	
C8	0.4822 (12)	0.7071 (5)	0.5791 (4)	0.0727 (13)	
H8	0.4856	0.6330	0.5393	0.087*	
C9	0.3510 (13)	0.8229 (7)	0.5349 (5)	0.0923 (17)	
H9A	0.3435	0.8991	0.5725	0.111*	
H9B	0.2637	0.8304	0.4653	0.111*	

### Atomic displacement parameters (Å^2^)

**Table:**

	*U*^11^	*U*^22^	*U*^33^	*U*^12^	*U*^13^	*U*^23^
Br1	0.0653 (3)	0.0414 (3)	0.0810 (4)	0.01520 (18)	−0.0026 (2)	−0.0228 (2)
N1	0.0438 (15)	0.0317 (14)	0.0475 (15)	0.0066 (11)	0.0071 (11)	−0.0044 (11)
C1	0.0459 (18)	0.0326 (17)	0.0504 (19)	0.0017 (14)	−0.0002 (14)	−0.0063 (14)
O1	0.0569 (15)	0.0348 (13)	0.0660 (16)	0.0104 (11)	0.0037 (12)	−0.0068 (11)
N2	0.0450 (15)	0.0336 (14)	0.0549 (17)	0.0030 (12)	0.0027 (13)	−0.0028 (12)
C2	0.0404 (17)	0.0413 (18)	0.0519 (19)	0.0003 (14)	−0.0030 (14)	−0.0091 (15)
N3	0.0468 (16)	0.0506 (18)	0.0585 (18)	0.0027 (13)	0.0070 (13)	−0.0084 (14)
C3	0.0443 (19)	0.056 (2)	0.060 (2)	0.0073 (16)	0.0025 (16)	−0.0189 (18)
C4	0.0459 (18)	0.0427 (18)	0.056 (2)	0.0057 (14)	−0.0047 (15)	−0.0181 (15)
C5	0.0457 (18)	0.0352 (17)	0.0512 (19)	0.0023 (14)	−0.0001 (14)	−0.0089 (14)
C6	0.0383 (16)	0.0344 (16)	0.0464 (18)	0.0013 (13)	−0.0010 (13)	−0.0095 (13)
C7	0.051 (2)	0.042 (2)	0.072 (2)	−0.0067 (16)	0.0028 (18)	0.0043 (17)
C8	0.101 (4)	0.058 (3)	0.051 (2)	−0.010 (2)	0.009 (2)	0.000 (2)
C9	0.094 (4)	0.102 (5)	0.068 (3)	−0.005 (3)	−0.007 (3)	0.014 (3)

### Geometric parameters (Å, °)

**Table:**

Br1—C4	1.905 (4)	C3—H3	0.9300
N1—C1	1.367 (4)	C4—C5	1.387 (5)
N1—C6	1.388 (4)	C5—C6	1.361 (5)
N1—H1	0.8600	C5—H5	0.9300
C1—O1	1.228 (4)	C7—C8	1.498 (7)
C1—N2	1.379 (5)	C7—H7A	0.9700
N2—C2	1.380 (5)	C7—H7B	0.9700
N2—C7	1.465 (5)	C8—C9	1.286 (8)
C2—N3	1.315 (5)	C8—H8	0.9300
C2—C6	1.406 (5)	C9—H9A	0.9300
N3—C3	1.351 (5)	C9—H9B	0.9300
C3—C4	1.381 (6)		
			
C1—N1—C6	110.1 (3)	C6—C5—C4	115.0 (3)
C1—N1—H1	124.9	C6—C5—H5	122.5
C6—N1—H1	124.9	C4—C5—H5	122.5
O1—C1—N1	127.3 (3)	C5—C6—N1	134.3 (3)
O1—C1—N2	126.0 (3)	C5—C6—C2	119.5 (3)
N1—C1—N2	106.8 (3)	N1—C6—C2	106.3 (3)
C1—N2—C2	109.5 (3)	N2—C7—C8	112.6 (3)
C1—N2—C7	124.0 (3)	N2—C7—H7A	109.1
C2—N2—C7	126.4 (3)	C8—C7—H7A	109.1
N3—C2—N2	126.4 (3)	N2—C7—H7B	109.1
N3—C2—C6	126.3 (3)	C8—C7—H7B	109.1
N2—C2—C6	107.3 (3)	H7A—C7—H7B	107.8
C2—N3—C3	114.0 (3)	C9—C8—C7	124.4 (5)
N3—C3—C4	123.0 (3)	C9—C8—H8	117.8
N3—C3—H3	118.5	C7—C8—H8	117.8
C4—C3—H3	118.5	C8—C9—H9A	120.0
C3—C4—C5	122.2 (3)	C8—C9—H9B	120.0
C3—C4—Br1	118.6 (3)	H9A—C9—H9B	120.0
C5—C4—Br1	119.2 (3)		

### Hydrogen-bond geometry (Å, °)

**Table:**

*D*—H···*A*	*D*—H	H···*A*	*D*···*A*	*D*—H···*A*
N1—H1···O1^i^	0.86	1.95	2.798 (4)	168

Symmetry codes: (i) −*x*, −*y*+1, −*z*+2.
